# Stereotactic body radiation therapy as an effective local treatment for advanced hepatocellular carcinoma patients with inferior vena cava and right atrial tumor thrombus

**DOI:** 10.1186/s12876-022-02556-8

**Published:** 2022-11-08

**Authors:** Dan Zhang, Qian Li, Dong Li, Jun Jia, JunQiang Ding, Jing Sun, Xuezhang Duan

**Affiliations:** Department of Radiation Oncology, The Fifth Medical Center of PLA General Hospital, No. 100 Xi Si Huan Middle Road, Fengtai District, Beijing, 100039 China

**Keywords:** SBRT, Advanced HCC, IVCTT, RATT, Targeted therapy, Immunotherapy

## Abstract

**Background:**

The aim of our study was to evaluate the curative effect and safety of stereotactic body radiation therapy (SBRT) in treating hepatocellular carcinoma (HCC) patients with inferior vena cava (IVCTT) and right atrial tumor thrombus (RATT).

**Methods:**

This retrospective study included fifteen advanced HCC patients with IVCTT and RATT who were treated with SBRT between 2013 and 2020. The prescribed dose delivered to the tumor was 45–50 Gy/7–10 fx. We report their treatment responses according to survival time and toxicities.

**Results:**

For these patients, the median follow-up time was 15 months (2–52 months). Local tumor control rates of the treated area were 80% at the time of death or at the last follow-up. The 6-month, 12-month, 18-month and 24-month OS rates were 80.0%, 60.0%, 33.3% and 26.7%, respectively. None of these patients died from the toxicity outcomes and complications of SBRT.

**Conclusion:**

SBRT is an effective option for advanced HCC patients with IVCTT and RATT.

Hepatocellular carcinoma (HCC) is the sixth most common cancer worldwide and the third most common cause of cancer-related death [1]. As the disease progresses, a small number of patients develop inferior vena cava tumor thrombosis and right atrial extension, which may result in pulmonary embolism and cardiac tamponade and shorten the survival time of patients.

Previous studies have shown stereotactic body radiation therapy (SBRT) to be a safe treatment, and it also brings satisfactory prognosis, especially for patients with inoperable or recurrent HCC [2–4]. Moreover, SBRT can also be used as palliative care for prolonging the survival time of advanced patients, which includes patients with metastases or vascular invasion.

This retrospective study on prognosis in advanced HCC patients with inferior vena cava (IVCTT) and right atrial tumor thrombus (RATT) treated with SBRT provides some insights for clinical treatment.

## Materials and methods

### Patient selection

We conducted a retrospective review of fifteen HCC patients with IVCTT and RATT treated with SBRT at the Fifth Medical Center of Chinese PLA General Hospital between September 2013 and December 2020. All patients voluntarily received SBRT treatment and signed informed consent forms.

### Radiation therapy and follow-up study

All enrolled patients were administered stereotactic body radiation therapy (CyberKnife, Accuray, USA). The oncologist contoured the gross tumor volume (GTV, including inferior vena cava and right atrial tumor thrombus) and organs at risk (OARs, including the heart, lungs, liver, stomach and spinal cord, etc.). Planning target volume (PTV) expanded 3–5 mm of GTV and avoided the OARs. The prescribed dose delivered to the PTV was 45–50 Gy in 7–10 fractions. The normal tissue dose was within the dose constraints (Table 1), which refer to AAPM TG-101 report [5]. After treatment with SBRT, these patients were followed up every 3 months until June 2022. The follow-up items included laboratory results, chest and abdominal CT/MRI, brain CT/MRI, and PET-CT examination if necessary.

**Table 1:** Dose constraints for various critical organs and delivered dose during designing radiation therapy plans in this study

	Dose constraint during designing plans		Delivered dose in this study	
Serial tissue	Threshold dose (Gy)	Max point dose (Gy)	Delivered dose (Gy) Mean (range)	Delivered max point dose (Gy) Mean (range)
Heart (< 15 cc)	32 Gy	38 Gy	20.72 (16.58–24.02)	31.30 (26.16–36.72)
Lung (1000 cc/1500 cc)	13.5 Gy/12.5 Gy		9.47 (7.22–12.75)/ 7.40 (5.95–9.45)	
Liver (700 cc/mean dose)	21 Gy/22 Gy		16.54 (11.70–20.95)/ 18.15 (12.94–21.85)	
Stomach (< 10 cc)	18 Gy	32 Gy	16.23 (13.96–18.18)	24.80 (21.08–27.60)
Duodenum (< 5 cc/ < 10 cc)	18 Gy/12.5 Gy	32 Gy/32 Gy	15.91 (14.61–17.19)/ 10.47 (9.33–11.78)	20.75 (18.63–23.14)
Spinal cord (< 0.35 cc)	23 Gy	30 Gy	20.27 (18.47–23.49)	22.84 (19.86–26.22)
Trachea and large bronchus (< 4 cc)	16.6 Gy	40 Gy	9.14 (6.39–12.58)	24.92 (21.44–30.09)
Bronchus-smaller airways (< 0.5 cc)	21 Gy	33 Gy	15.45 (12.74–19.25)	20.77(17.99–26.10)

## Results

**Fig. 1:**
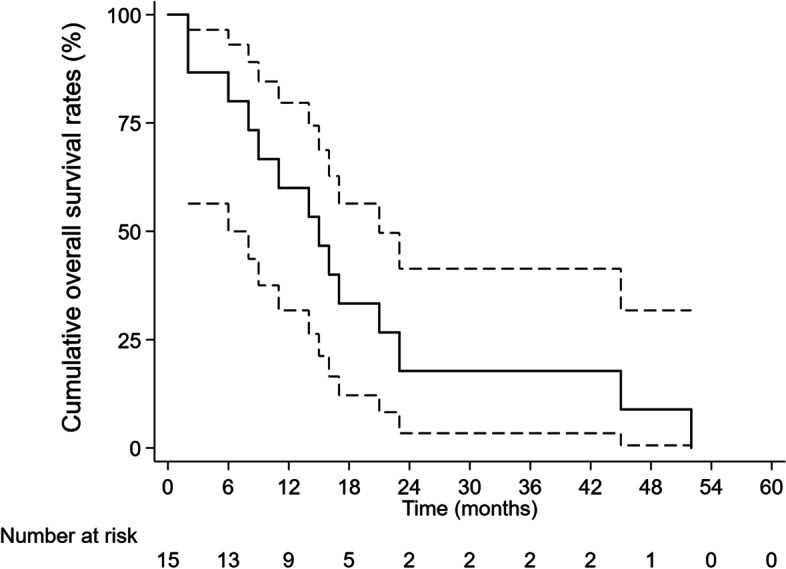
The overall survival curve in fifteen patients

**Fig. 2:**
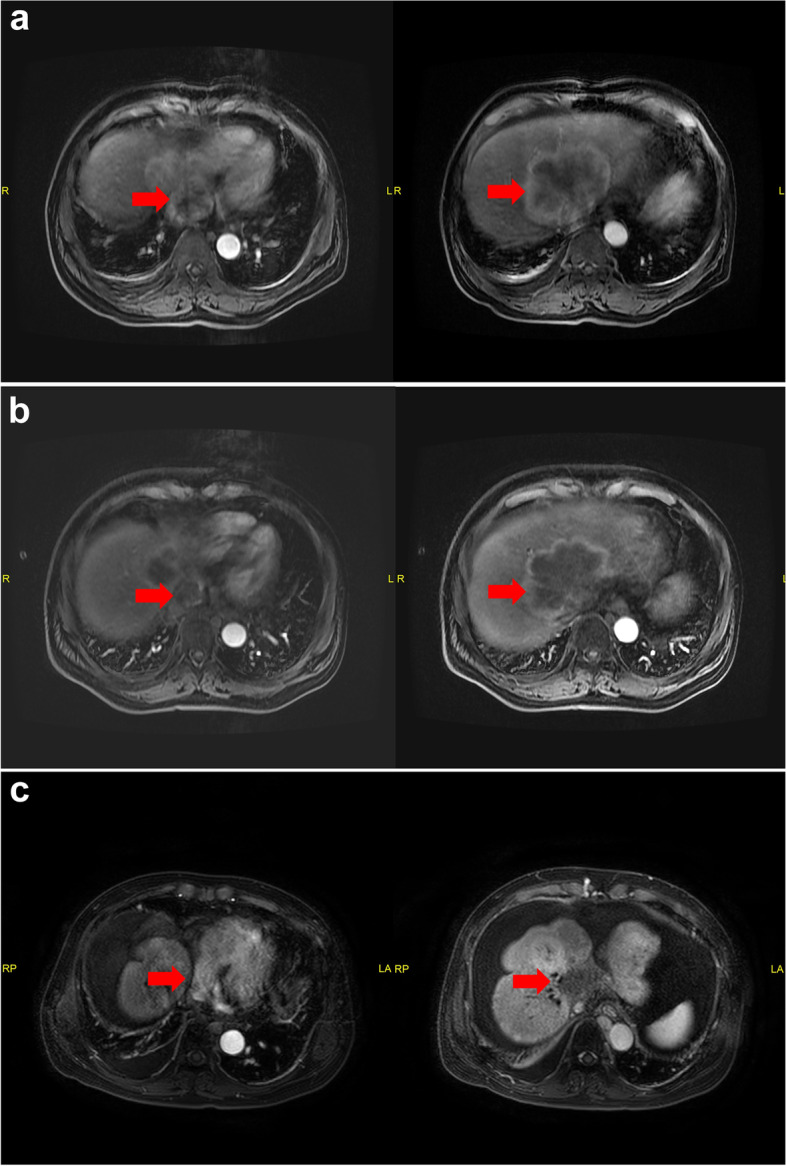
The classic patient (Case 9) with IVCTT and RATT received SBRT. January 2018: The primary upper-abdominal MRI scan with lesions **a** MRI scan in May 2018 **b** and March 2022 **c** after SBRT. The patient died of infectious shock due to hepatic failure in May 2022

The median age was 51 years old (32–69 years old). There were 14 hepatitis B patients (93.3%) and 1 hepatitis C patient (6.7%). Ten patients had Child–Pugh A classification (66.7%) and 5 had Child–Pugh B classification (33.3%). Two patients were ALBI grade 1 (13.33%), 9 were ALBI Grade 2 (60.0%).and 4 were ALBI Grade 3 (26.70%). The AFP level ranged from 12 ng/ml to 39,231 ng/ml. All patients previously received other treatments for liver lesions, such as TACE, targeted therapy or SBRT. The median follow-up period was 15 months (2–52 months). During the follow-up period after SBRT, we found that 11 patients’ GTV lesions shrank, 3 enlarged and 1 showed no change. By June 2022, 14 patients had died (Table 2). The 6-month, 12-month, 18-month and 24-month OS rates were 80.0%, 60.0%, 33.3% and 26.7%, respectively (Fig. 1). A classic case is shown in Fig. 2.

**Table 2:** Baseline characteristics, treatment details, and follow-up of the study population

				The details of patients before SBRT						Treatment protocols		Follow-up and review outcome		Survival outcome		
No	Gender	Age	AFP (ng/ml)	With virus hepatitis infection (B/C/None)	Child–Pugh Classification	ALBI grade	ECOG PS score	Previous treatments	Extrahepatic metastasis	Prescribed doses	Combination treatments	Other active lesions (time to progression)	Targeted lesion progression after SBRT (Y/N)	Follow-up period (months)	Living/dead	Cause of death
1	Male	51	143	Hepatitis B virus infection	A6	2	1	Liver: TACE, Lenvatinib	1. Lung 2.Mediastinal lymph node metastasis	50 Gy/10fx	None	1. Lung: Progression (uncontrolled) 2. Mediastinal lymph node metastasis: Progression (uncontrolled) 3. Liver: Progression (3 months)	N	8	Dead	Respiratory failure due to lung metastases
2	Male	54	2837	Hepatitis B virus infection	A6	2	1	Liver: Hepatic Resection, Sorafenib	None	45 Gy/9fx	None	Lung: New lesions (13 months)	N	15	Dead	Infectious shock due to hepatic failure
3	Male	62	12	Hepatitis B virus infection	B7	3	1	Liver: Radiofrequency ablation, TACE	1. Lung 2. Kidney	49 Gy/7fx	None	1. Lung: uncontrolled 2. Liver: progression (1 month) 3. Kidney: uncontrolled	Y	6	Dead	Hepatic encephalopathy due to hepatic failure
4	Male	48	4856	Hepatitis B virus infection	A6	2	1	Liver: TACE	Retroperitoneal lymph node metastasis	45 Gy/9fx	Sorafenib	1.Retroperitoneal lymph node metastasis: uncontrolled 2. Liver: Progression (3 months)	N	16	Dead	Infectious shock due to hepatic failure
5	Male	50	956	Hepatitis B virus infection	A6	2	0	Liver: Hepatic resection	None	50 Gy/10fx	Lenvatinib	Liver: Progression (7 months)	N	14	Dead	Unknown causes
6	Female	46	1517	Hepatitis B virus infection	A6	2	0	Liver: TACE	1. Lung 2. Retroperitoneal lymph node metastasis	50 Gy/10fx	None	1. Lung: uncontrolled 2. Retroperitoneal lymph node metastasis: No change 3.Liver: Progression (1 months)	N	9	Dead	Infectious shock due to hepatic failure
7	Male	47	29	Hepatitis B virus infection	B7	3	1	Liver: Radiofrequency ablation, TACE	1. Lung 2. Intracranial metastases	50 Gy/10fx	Lenvatinib	1. Liver: uncontrolled 2. Lung: uncontrolled 3. intracranial metastases: uncontrolled	Y	2	Dead	Renal failure due to hepatic failure
8	Male	45	189	Hepatitis B virus infection	A5	1	0	Liver: SBRT	Lung	49 Gy/7fx	Sorafenib	1. Lung (uncontrolled) 2. Bone: New lesions (9 months), with radiotherapy as additional treatment for pain relief	N	17	Dead	Respiratory failure due to lung metastases
9	Male	69	1210	Hepatitis B virus infection	A5	2	0	Liver: SBRT, TACE	None	49 Gy/7fx	Sorafenib	Lung: New lesions (21 months), with Lenvatinib and toripalimab as additional treatment	N	52	Dead	Infectious shock due to hepatic failure
10	Male	58	2435	Hepatitis B virus infection	A5	1	1	Liver: Hepatic resection, Sorafenib	None	50 Gy/10fx	Lenvatinib and toripalimab	Liver: Progression (1 months)	N	45	Dead	Upper gastrointestinal bleeding
11	Male	32	323	Hepatitis B virus infection	B7	2	1	Liver: TACE, Sorafenib	Mesenteric lymph node metastasis	49 Gy/7fx	Lenvatinib	1. Mesenteric lymph node metastasis: uncontrolled 2. Liver: progression (1 month)	Y	2	Dead	Upper gastrointestinal bleeding
12	Female	57	39,231	Hepatitis B virus infection	B7	3	0	Liver: TACE	Lung	45 Gy/9fx	Toripalimab	Lung: New lesions (12 months)	N	23	Dead	Upper gastrointestinal bleeding
13	Male	64	2139	Hepatitis C virus infection	A6	2	1	Liver: SBRT, TACE	Retroperitoneal lymph node metastasis	45 Gy/9fx	Lenvatinib and toripalimab	Retroperitoneal lymph node metastasis: no change Liver: New lesion (12 months)	N	21	Dead	Upper gastrointestinal bleeding
14	Male	48	176	Hepatitis B virus infection	A6	2	1	Liver: SBRT, TACE	None	45 Gy/9fx	Toripalimab	Lung: New lesions (21 months) Liver: New lesion (15 months), add TACE	N	21	Living	
15	Female	68	5679	Hepatitis B virus infection	B7	3	0	Liver: TACE	None	50 Gy/10fx	Lenvatinib	Liver: Progression (5 months) Lung: New lesion (2 months), add toripalimab	N	11	Dead	Unknown causes

### Toxicity and complications

All patients in our study had liver cirrhosis of different degrees and portal hypertension, which could lead to esophageal and gastric varices. Three patients died of upper gastrointestinal bleeding within two years of SBRT. Patients 11 received emergency gastroscopy for gastrointestinal bleeding, which was confirmed to be gastric vein rupture and obvious varicose manifestation at the bleeding site. Patient 12 and 13 received gastroscopy regularly and no ulcer was found, but obvious varicose manifestations was observed. By reviewing their treatment plans, 10 cc and maximum point dose to stomach of patient 12 were 17.60 Gy and 25.50 Gy, respectively. Those of patient 13 were 16.50 Gy and 25.64 Gy, respectively. Both plans were under dose constraints value. In addition, these two patients died over 20 months after SBRT. Thus we concluded that their gastrointestinal bleeding was related to liver cirrhosis.

The complications related to liver failure occurred in six patients. We carefully reviewed the SBRT plan and confirmed that 700 cc normal liver dose and mean liver dose of these six cases were 16.08 (11.70–19.20) Gy and 18.12 (12.94–21.67) Gy, respectively. In addition, RILD usually occurs within 3 months after radiotherapy. All these six patients died over 6 months after SBRT [6]. In addition, the intrahepatic lesions progressed in all of these patients. Therefore, it was considered that the main cause of death is disease progression rather than toxicities of treatment.

During and after SBRT, no toxicities of Grade ≥ 3 were observed in lungs, esophagus and trachea. It is worth noting that none of the patients in this study had cardiac-related complications or malignant pericardial effusion. We considered that SBRT is treated with noncoplanar radiation, and more radiation angles will better protect the normal organs.

## Discussion

Advanced HCC always has vascular invasion, most commonly the portal vein and hepatic veins. Previous studies showed that the incidence of HCC with IVCTT was approximately 3.8% [7], while RATT occurred in approximately 2% of the cases [8]. In general, the median survival time of advanced HCC patients with IVCTT has been shown to be less than 6 months, with no patients surviving beyond 2 years [9], which is even worse in those with RATT. In recent years, the treatment modalities for advanced HCC patients with IVCTT and/or RA include thrombectomy, transhepatic arterial chemotherapy embolization (TACE), targeted therapy and immunotherapy (checkpoint inhibitor of the programmed cell death protein-1, PD-1 inhibitor). However, a few clinical studies have been reported, and the sample size of the studies was small.

Tomita K [10] conducted a study of three patients with thrombi in the inferior vena cava, right atrium and phrenic vein who underwent resection. The results showed that the microscopic surgical margins of the combined resected diaphragms were positive in all cases, and the respective overall postoperative survival was 98.0, 38.9, and 30.9 months. The patients died due to liver cirrhosis, acute heart failure, and hepatocellular carcinoma. Chai Hong Rim reported a meta-analysis and systematic review, and his results showed pooled 1- and 2-year OS rates were 53.6% and 36.9%, respectively [11], which were better than ours. We found that 60.0% (9/15) of patients in our study also had other active metastases, such as lung and/or lymph nodes metastases; therefore, their treatments were mainly palliative care, which may affect their prognosis. In our study, patients 9 and 10 had active tumors located in blood vessels, and their survival times were all more than 3 years.

It is necessary to carry out a multicenter prospective study to explore the efficacy of IVCTT and RATT in HCC patients. The results of our study provide some ideas for clinical work, but further research and confirmation are still needed.

## Data Availability

All data generated or analyzed during this study are included in this published article.
